# Adaptation of the Food Literacy (FOODLIT) Tool for Turkish Adults: A Validity and Reliability Study

**DOI:** 10.3390/nu16193416

**Published:** 2024-10-09

**Authors:** Yasemin Ertaş Öztürk, Sevtap Kabalı, Yasemin Açar, Duygu Ağagündüz, Ferenc Budán

**Affiliations:** 1 Department of Nutrition and Dietetics, Ondokuz Mayıs University, 55200 Samsun, Türkiye; sevtap.kkurtaran@omu.edu.tr (S.K.); dytyaseminacar@gmail.com (Y.A.); 2 Department of Nutrition and Dietetics, Gazi University, 06490 Ankara, Türkiye; duyguturkozu@gazi.edu.tr; 3 Institute of Physiology, Medical School, University of Pécs, H-7624 Pécs, Hungary

**Keywords:** food literacy, sustainability, validity and reliability, nutrition, Turkish adults, measurement tool

## Abstract

Background: Food literacy is associated with sustainable food systems and encourages individuals to adopt healthy eating habits. However, there is no validated method that can be used to measure food literacy related to sustainable food systems of Turkish adults. This research aimed to assess the validity and reliability of the Turkish adaptation of the “Food Literacy (FOODLIT) Tool” for Turkish adults. Methods: The study involved 328 people aged 19 to 58 years. The FOODLIT-Tool is a five-point Likert-type scale consisting of 24 items and five factors (“culinary competencies”, “production and quality”, “selection and planning”, “environmentally safe” and “origin”). Results: The Cronbach’s alpha coefficient was applied to assess internal consistency reliability, showing an excellent scale coefficient of 0.927. The model was evaluated with a confirmatory factor analysis (CFA). The findings of the CFA suggested that the fit indices were acceptable (χ^2^/df = 1.257, comparative fit index: 0.991, goodness-of-fit index: 0.977, normed fit index: 0.990 and root mean error of approximation: 0.028). Furthermore, there was a positive relationship between the FOODLIT-Tool score and the “Sustainable and Healthy Eating Behaviors Scale” (SHEB) score (r = 0.518, *p* < 0.001). Conclusion: Our study shows that the Turkish version of the FOODLIT-Tool integrated with sustainable food systems is a valid and reliable measurement tool for assessing the food literacy of Turkish adults.

## 1. Introduction

Food literacy is a factor that has recently gained increasing importance and encourages healthy eating habits. According to Vidgen and Galleos (2014), food literacy is “the acquisition of the knowledge, skills and behaviors necessary to plan, manage, select, prepare and eat foods to determine nutrient needs and consumption. In other words, it is the structure that empowers individuals and communities to maintain diet quality” [1]. Food literacy has many determinants and influencing factors. These are essentially listed as food/health choices, skills and behaviors, culture, emotions, knowledge and food systems [2]. All the components mentioned above provide individuals and societies with the freedom to choose food and provide a critical perspective on food choice. On the other hand, inadequacy in food literacy may lead to a lack of information about the process of food from farm to fork [3,4]. Inadequate literacy, together with easy access to unhealthy foods in the food supply chain and the loss of culinary skills during food preparation, may lead to inappropriate consumer behaviors in the food system [4,5].

In recent years, there has been a global change in food systems due to individual and environmental factors [6]. More than half of the United Nations’ 17 Sustainable Development Goals to be achieved by 2030 are related to health, environment and food systems [7]. Studies have shown that the production, processing, packaging, distribution and consumption steps of food are responsible for one-third of total greenhouse gas emissions [8]. In this context, a sustainable food system should meet the needs of individuals from a health and nutrition perspective and ensure access to healthy and safe food [9,10]. Similar to this trend, food literacy encompasses a wide range of issues, from individual and public health to the food system and its interaction with the environment. Recent studies on food literacy suggest that the term should include multifaceted decision-making concepts related to food and the food system. In this respect, the sustainable food system can be integrated into the conceptual framework of food literacy, and issues such as sustainability and food safety can be addressed in the process from food production to consumption [11,12,13,14].

Rosas et al. conducted the Food Literacy Project (FOODLIT-PRO) [15], which aimed to integrate the sustainable food system and food literacy into the conceptual framework and subsequently revealed the Food Literacy Wheel (FLW) [16]. With this project, the definition, determinants and influential factors of food literacy have been comprehensively presented. In line with sustainable food systems, the FLW includes health, nutrition, cultural, social, sustainability, industry, policy, learning and psychological contexts as influential factors. In addition, the wheel includes a series of stages such as food selection, purchasing, preservation, planning and culinary skills. In this context, in addition to the assessment of consumers’ food literacy status, the need to evaluate the heterogeneous factors and determinants of food literacy has emerged [16,17]. The need to assess sustainable food systems that integrate all health, environmental, social and economic perspectives emphasizes both the food literacy of consumers and the importance of the food supply chain [18]. In recent years, instruments assessing consumers’ food literacy have been developed [19,20]. However, there is no validated method that can be used to measure food literacy related to sustainable food systems of Turkish adults. In this study, we aimed to assess the validity and reliability of the “Food Literacy Tool (FOODLIT-Tool)” for Turkish adults.

The importance of the validity and reliability of the FOODLIT-Tool for Turkish adults can be listed as follows: (1) In addition to measuring consumers’ food literacy, this tool takes into account different perspectives (environmentally safe, production and quality, selection and planning, culinary skills, origin). (2) This tool provides targets for understanding and intervention in the food supply chain. (3) This tool focuses not only on the level of knowledge about food but also on sustainability, meal planning, food safety and food preparation skills. We believe that this study will contribute to future studies on the assessment of food literacy related to sustainable food systems.

## 2. Materials and Methods

### 2.1. Adaptation to Turkish Language and Content Validity

The original instructions and items of the FOODLIT-Tool (Supplementary Material S1) underwent translation into Turkish and subsequent back translation into English in accordance with the suggested recommendations [21]. Two independent academicians with advanced English and a linguist translated the tool to Turkish. Afterward, the Turkish version of the tool was back translated into English by two independent nutrition experts who had never seen the original English version of the tool. After the translation of the tool was finalized, Turkish form was sent to ten nutrition experts and they gave their opinions on content. Experts rated the items and the item-based content validity ratio and content validity index (CVI) for each factor, and total was calculated. The CVI values were >0.800, and it was above the critical value [22].

A pilot study was conducted on a sample of 17 people to examine the acceptability and understanding of the items. Participants studied the tool and were invited to express their comments on each item. Minor suggestions (e.g., synonym alteration) for increasing the understanding were accepted and considered for two items. The final Turkish version of the tool is presented in Supplementary Material S2.

### 2.2. Participants and Data Collection

This study was conducted on 328 participants (73.5% women) from December 2023 to February 2024. Data were obtained through the Google Forms web platform. Any adults (19–65 years) who speak Turkish language were included in the study. People with any psychiatric disorder, following a special dietary regimen, and pregnant or lactating women were not included. Adherence to the inclusion and exclusion criteria was achieved through initial inquiries regarding the relevant criteria. The presence of psychiatric illness was assessed based on whether it was diagnosed by a physician. The study questionnaire was administered to participants at a ratio above 1:20 (500 subjects), providing equal representation of both sexes; however, in the end, the data from all persons who volunteered to participate were analyzed. Mean age of the participants was 26.6 ± 8.36 years; 62.6% of them had undergraduate or higher educational degree, 71.6% were single, and 22.6% were low-income status.

The research received approval from the Ondokuz Mayıs University Clinical Research Ethics Committee (decision number: 2023/357-B.30.2.ODM.0.20.08/554, date: 8 November 2023), following the principles of the Helsinki Declaration.

### 2.3. Measures

#### 2.3.1. Food Literacy Tool

The FOODLIT-Tool was created by Rosas et al. [18] through a qualitative investigation of the definition, determinants and influential aspects of food literacy [15]. The construction of a conceptual and empirical framework encompasses the fundamental knowledge, competencies and behaviors connected to food. It also considers the factors that facilitate or hinder these aspects, as well as the various areas of interaction that aim to address broader concerns related to global sustainability in food systems [16] as part of the FOODLIT-PRO [15]. The tool contains 24 items, five factors (F1: Culinary competencies, F2: Production and quality, F3: Selection and planning, F4: Environmentally safe, F5: Origin) and Likert-type scoring system (0—never/totally disagree, 1—sometimes/disagree, 2—frequently/agree, 3—always/totally agree). Each item is scored between 0 and 3 points. The total score and the scores of the sub-factors are obtained by totaling the relevant items. The original Cronbach’s alpha value is 0.831.

#### 2.3.2. Sustainable and Healthy Eating Behaviors Scale

“Sustainable and Healthy Eating Behaviors” (SHEB) Scale was originally developed by Żakowska-Biemans et al. [23] and adapted into Turkish by Köksal et al. [24]. The Turkish version of the scale consists of 32 items and seven factors that are SHEB_F1: “Quality labels (regional and organic)”, SHEB_F2: “Seasonal food and avoiding food waste”, SHEB_F3: “Animal welfare”, SHEB_F4: “Meat reduction”, SHEB_F5: “Healthy and balanced diet”, SHEB_F6: “Local food” and SHEB_F7: “Low fat”. Likert-type scale was used and scored 1 to 7. Respondents were asked to rate each item as ‘never’, ‘very rarely’, ‘rarely’, ‘sometimes’, ‘often’, ‘very often’ or ‘always’. Higher scores obtained indicate that the individual had the characteristics evaluated by the relevant factors. The Cronbach’s alpha value is 0.912.

### 2.4. Statistical Analysis

Internal consistency reliability was assessed using Cronbach’s alpha coefficients, which uses a polychronic correlation matrix since it is more suitable than the Pearson correlation for ordinal data [25]. In order to test the constant validity of the FOODLIT-Tool, a confirmatory factor analysis (CFA) was conducted. Since the original tool was developed based on a framework and the items were parts of the conceptual and empirical framework with a mixed methodology [16], no exploratory factor analysis was conducted. The model was tested using the diagonally weighted least squares (DWLS) procedure. In order to determine whether the model had a good fit, the following fit indices were examined: chi-square/df, Comparative Fit Index (CFI), Good of Fit Index (GFI), Tucker–Lewis Fit Index (TLI), the Non-Normed Fit Index (NNFI), the Incremental Fit Index (IFI), the adjusted goodness of fit index (AGFI), the Standardized Root Mean Square Residual (SRMR) and the Root Mean Square Error of Approximation (RMSEA). We accepted chi-square/df < 2.0, CFI, GFI, TLI, NNFI, IFI values of ≥0.90, AGFI > 0.85 and RMSEA and SRMR ≤ 0.10 as cut-off values [26,27,28,29]. Also, we examined the concurrent validity of the tool by performing a correlation analysis between the FOODLIT-Tool and SHEB Scale total and sub-factors. Statistical findings were obtained using IBM SPSS 28 and R software (version 4.4.1), and the type-1 error level was set at α = 0.05.

## 3. Results

The model was evaluated with subjects who responded to every item on the scale (n = 328) with a participant-to-item ratio of >10:1. The skewness and kurtosis results showed that the item answers exhibited a normal univariate distribution, with acceptable ranges for skewness and kurtosis being −2 to +2 and −7 to +7, respectively. After removing ten outliers, the multivariate normality was established (Mahalanobis distance maximum value = 48.600, *p* < 0.001).

**Figure 1:**
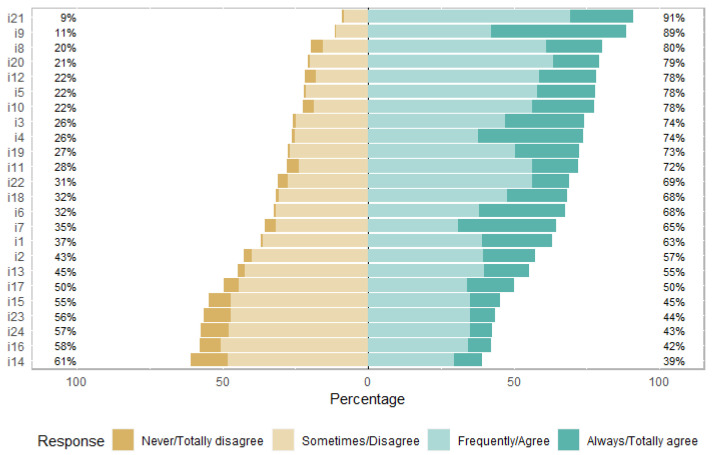
Responses to the FOODLIT-Tool items.

The answers to the items are shown in Figure 1. The majority of the participants (91%) totally agreed with i21, which is “I am aware of the time of year of each food”. The item most disagreed (61%) with was i14, which is “I control the calories and/or other nutritional characteristics of the food I eat daily”.

The Cronbach’s alpha coefficients of total and factors are presented in Table 1. Accordingly, the total and sub-factor scores are reliable.

**Figure 2:**
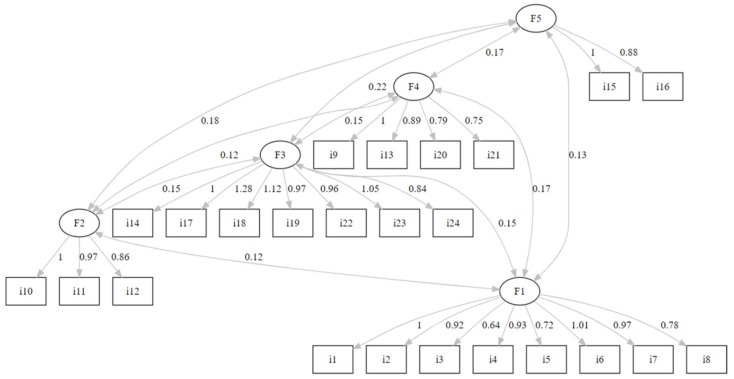
Confirmatory factor analysis for the coefficients of the model.

The goodness of fit index for the CFA analysis (five factors and 24 items) is presented in Table 2, and the coefficients for the full model are presented in Figure 2.

**Figure 3:**
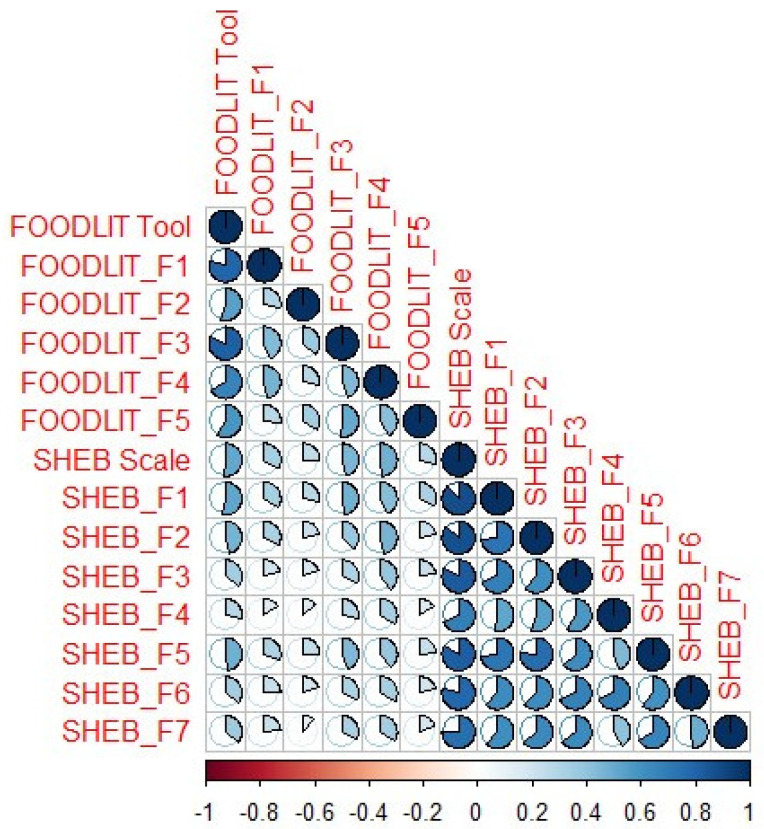
Correlations between the FOODLIT-Tool and the SHEB Scale. The ratio of the pies represents the correlation of the coefficient levels. All relationships were statistically significant (*p* < 0.05). FOODLIT: Food literacy; SHEB: Sustainable and Healthy Eating Behaviors; SHEB_F1: “Quality labels (regional and organic)”; SHEB_F2: “Seasonal food and avoiding food waste”; SHEB_F3: “Animal welfare”; SHEB_F4: “Meat reduction”; SHEB_F5: “Healthy and balanced diet”; SHEB_F6: “Local food”; SHEB_F7: “Low fat”.

To assess concurrent validity, correlational analysis between the FOODLIT-Tool and the SHEB Scale was conducted and is reported in Figure 3. Positive correlations were observed between the FOODLIT-Tool factors and the SHEB Scale sub-factors (*p* < 0.05). The strongest associations were between the FOODLIT-Tool and the SHEB Scale (r = 0.518, *p* < 0.001) and the FOODLIT-Tool and the SHEB_F1 factor (r = 0.528, *p* < 0.001).

## 4. Discussion

Food literacy refers to an individual’s capacity to understand, perceive and engage with the intricate food system over the course of their lifetime. Food literacy encompasses an individual’s understanding, abilities, beliefs and behaviors related to eating [30]. Enhancing food literacy not only improves individualized nutrition, health and wellness but also enables individuals to understand the impact of their food choices on the environment and how their food selections affect others [30,31]. The macro perspective of food literacy separates it into three primary areas: food, nutrition and health; agriculture, environment and ecology; and social development and equity. Food literacy is viewed as a component of food, health, sustainable environments and social equality [32].

Recently, there has been an increasing interest in examining the connection between food literacy and its impact on health outcomes. Individuals with a deficiency in basic knowledge about food are at a higher risk of developing ailments such as obesity, chronic heart disease, diabetes and cancer [33,34]. People with low food literacy may be unaware of how many calories are in a serving of their meal, as well as how much salt or sugar it contains [35]. They may have a limited understanding of the potential advantages or disadvantages of the dietary components. They may lack knowledge about the appropriate dietary choices for their age or health condition, leading to uncertainty about which foods they should consume more or less frequently [34,36]. In this context, it is important to increase the food literacy awareness levels of individuals.

Sustainable diets are defined by the Food and Agriculture Organization as follows: “Sustainable Diets are those diets with low environmental impacts which contribute to food and nutrition security and to healthy life for present and future generations. Sustainable diets are protective and respectful of biodiversity and ecosystems, culturally acceptable, accessible, economically fair and affordable; nutritionally adequate, safe and healthy; while optimizing natural and human resources” [37]. These diets aim to improve food security, promote health for current and future generations, and mitigate environmental impact [38].

Food literacy is a key aspect of sustainability since it plays a role in promoting social, economic and environmental sustainability. Food literacy cultivates an understanding of the connection between food consumption and the environment. Possessing a high level of literacy can contribute to mitigating the impact of humans on the environment by making informed and sustainable dietary choices [39,40]. The outcomes of good food literacy prevent the loss of resources such as soil, water and energy at all stages of the food supply chain and reduce greenhouse gas emissions that cause climate change [4,41]. Cullen et al. [42] defined food literacy through the viewpoint of community food security as the acquisition of knowledge in three domains: (1) the impact of food on personal health and well-being; (2) the multifaceted nature of the food system encompassing societal, economic, cultural, environmental and political perspectives; and (3) the comprehensive food system, spanning from production to waste management. This notion underscores the necessity of cultivating knowledge, skills and habits pertaining to food to improve decision-making that benefits individuals and fosters a sustainable food system.

This study aimed to assess the validity and reliability of the Turkish adaption of the FOODLIT-Tool among the Turkish population. This study consisted of a total of 328 people. This scale is suitable for utilization among adult individuals. It is hypothesized that those working in various fields of healthcare, specifically nutrition and dietetics, could utilize this scale to enhance the advancement of nutritional knowledge, skills and behaviors. The number of scales in Türkiye available to assess adults’ food literacy is limited. This study is expected to fill a gap in the literature and lead to further research on food literacy. Various food literacy scales are utilized globally, such as the Food and Nutrition Literacy Instrument [19], the Self-Perceived Food Literacy Scale [43,44], the International Food Literacy Questionnaire [45] and the Food Literacy Behavior Checklist [46]. Participants with higher levels of food literacy consumed considerably more fruits, vegetables and fish than those with lower levels of food literacy. Furthermore, it was underlined that assessing the literacy levels of persons during the initial patient encounter and prior to providing dietary guidance will enhance the enhancement of nutritional well-being [19,43,44,45,46].

Our results showed that the Turkish version of the FOODLIT-Tool is valid and reliable, with 24 items and five factors. The factors are culinary competencies, production and quality, selection and planning, environmentally safe and origin. These factors are measured by a total of eight, three, seven, four and two items, respectively. The overall FOODLIT-Tool questionnaire has reliable internal consistency (Cronbach’s α = 0.927). The Cronbach’s α coefficients for the five factors (culinary competencies, production and quality, selection and planning, environmentally safe and origin) were 0.871, 0.883, 0.903, 0.763 and 0.866, respectively, which are higher than the original scale (0.54 to 0.73). The five-factor model (Figure 2) showed confirmatory factor analysis and an acceptable goodness of fit index (χ^2^/df = 1.257, CFI = 0.991, GFI = 0.977, TLI = 0.990, NNFI = 0.990, IFI = 0.991, AGFI = 0.971, SRMR = 0.061 and RMSEA = 0.028) (Table 2). In the original scale, Rosas et al. found fit indices as χ^2^/df = 3.958, SRMR = 0.055, RMSEA = 0.055, CFI = 0.907, and GFI = 0.917 [18]. We accepted chi-square/df < 2.0, CFI, GFI, TLI, NNFI, IFI values of ≥0.90, AGFI > 0.85, and RMSEA and SRMR ≤ 0.10 as cut-off values [26,27,28,29]. It can be seen that the fit indices we found as a result of the analyses performed in this study are within the accepted range and have similar values to the original scale. These results show that the Turkish version of the FOODLIT-Tool is a valid and reliable scale in determining food literacy.

The answers to the items are shown in Figure 1. Participants did not care about controlling the calories and nutritional characteristics of the food they ate daily. Individuals with an obsession with healthy eating, such as orthorexia nervosa, may have conditions such as controlling the calories of the foods consumed and being anxious about healthy food consumption. These conditions develop with the desire for a healthy diet, and concerns about health, nutrition and food quality are at the forefront [47,48].

We used the SHEB Scale, which was adapted into Turkish by Köksal et al. [24], indicating that the factors of the SHEB Scale are similar to those of the FOODLIT-Tool. Both scales prioritize promoting the consumption of seasonal and regional foods while mitigating food waste within the framework of sustainability. The second factor of the SHEB Scale, titled “seasonal food and avoiding food waste”, includes the following items related to food waste: “I don’t waste food”, “I reuse leftovers from food”, “I buy regional food”, “I eat seasonal fruits and vegetables”, “In season, I shop at farmer’s markets”, “I avoid sugary drinks”, “I limit my salt usage”. In the FOODLIT-Tool, the items related to food waste are given as “I buy local/national trade products to support local/national business”, “I eat food according to its seasonality”, “I am aware of the time of year of each food”. The two scales are similar in that they emphasize issues such as consuming seasonal foods, supporting local/national products/producers and not wasting food waste and recycling it. We found positive relationships between the FOODLIT-Tool factors and the SHEB Scale sub-factors. All relationships were statistically significant (*p* < 0.05). When the mean values of the SHEB Scale’s sub-factors were compared, the quality labels factor (factor 1), which includes regional and organic items, had the highest value [24]. These findings show that quality is an important criterion for creating consumer awareness and food literacy in choosing local products that are healthier, unprocessed and contain fewer preservatives. A few studies have explored the associations between food literacy and sustainable and healthy eating behaviors; Kabasakal-Cetin et al. [49] examined the impact of food literacy and sustainable eating behaviors on the intake of ultra-processed foods. They found that lower food literacy (β = −0.140, *p* = 0.004) and sustainable and healthy eating behaviors (β = −0.104, *p* = 0.032) predicted increased intake of ultra-processed foods. In another study, Mortaş et al. investigated the relationship between food and nutrition literacy and sustainable, healthy eating behaviors among young adults. They found that there were significant relationships between the SHEB Scale and the Food and Nutrition Literacy Instrument [48]. Our findings are consistent with these studies, showing that food literacy is associated with sustainability and healthy eating skills. Increased food literacy was positively connected with sustainable eating behavior in adults [20,50,51,52].

This study has highlighted a number of issues that affect both individual and public health and well-being, such as encouraging consumers to make food choices related to sustainability and health, environmental safety, origin, food supply chain and food safety, meal planning and culinary skills. We believe that the FOODLIT-Tool integrated with sustainable food systems is a useful and valid tool that can be used to comprehensively assess the food literacy levels of consumers in Türkiye. Furthermore, the FOODLIT-Tool can be used in longitudinal studies to assess changes in consumers’ food literacy over time. National policies can be developed to create educational programs for consumers based on the FOODLIT-Tool results and to eliminate deficiencies. In this context, in addition to providing information about the process from production to consumption of food, consumer education on sustainable food systems can be planned. The effectiveness of planned food-literacy education can be determined by using this tool to identify the aspects of the target groups that need to be improved. These food literacy components can be included in Good Manufacturing Practices to raise awareness of food producers at the national level. Thus, steps can be taken to encourage food producers and consumers to encourage the adoption of sustainable food production and consumption behaviors.

However, several limitations also need to be acknowledged. At first, we utilized self-reported health data and did not employ any screening tools to verify physiological abnormalities. The research predominantly focuses on women; subsequent studies should incorporate a greater representation of Turkish men, as their perspectives can differ.

## 5. Conclusions

The results of the study show that the Turkish adaptation of the FOODLIT-Tool is valid and reliable. We believe that this tool will serve as a reliable instrument for assessing the level of food literacy among individuals residing in Türkiye and subsequently uncover the influence of food literacy on culinary competencies, production and quality, selection and planning, environmental safety and origin of the food. Ultimately, it will provide assistance for future research endeavors aimed at determining the level of food literacy in comparable environments and age cohorts. Enhancing food literacy will provide individuals and communities with an understanding of sustainable food systems and will encourage maintaining healthy dietary patterns. We consider that it would be beneficial to evaluate food literacy for a healthier society, a clean environment and a sustainable agricultural supply. The education sector employees, food producers and policy makers can create this awareness through their co-operation.

## Figures and Tables

**Table 1:** Reliability of the factors and total tool.

Factors	Items	Item Number	Cronbach’s Alpha
F1: Culinary competencies	i1, i2, i3, i4, i5, i6, i7, i8	8	0.871
F2: Production and quality	i10, i11, i12	3	0.883
F3: Selection and planning	i14, i17, i18, i19, i22, i23, i24	7	0.903
F4: Environmentally safe	i9, i13, i20, i21	4	0.763
F5: Origin	i15, i16	2	0.866
Total	all items	24	0.927

**Table 2:** The goodness of fit index.

	χ^2^/df	CFI	GFI	TLI	NNFI	IFI	AGFI	SRMR	RMSEA
Model	1.257	0.991	0.977	0.990	0.990	0.991	0.971	0.061	0.028

## Data Availability

Dataset available upon request from the authors due to privacy.

## Supplementary Materials

The following supporting information can be downloaded at: https://www.mdpi.com/article/10.3390/nu16193416/s1. File S1: The original items of FOODLIT-Tool; File S2: Food Literacy Tool Turkish Version.
