# Supplementary material for: Adaptation of the Food Literacy (FOODLIT) Tool for Turkish Adults: A Validity and Reliability Study
Source: Nutrients. 2024 Oct 9;16(19):3416. doi: 10.3390/nu16193416 (PMC11478845; doi:10.3390/nu16193416)
Supplement: Supplementary file 1 [file nutrients-16-03416-s001.zip › Supplemantary Material File S1.pdf]

Supplemantary Material File S1. The original items of FOODLIT-Tool

Factors	Items
F1: Culinary competencies	
i1	I easily prepare everything that is necessary to make a meal.
i2	I combine different ingredients to create a suitable meal.
i3	I adapt recipes to be more to my taste.
i4	I use kitchen equipment and utensils (e.g., oven, blender) efficiently.
i5	I cook adequate meals with what I usually have at home.
i6	I cook in different ways (e.g., stewing, baking).
i7	I enjoy cooking.
i8	I have knowledge of different types of preservation (e.g., freezing, salting) suitable for different foods.
F2: Production and quality	
i10	I recognise the impacts of pesticides and/or herbicides on food.
i11	I know what organic products are (e.g., food grown without pesticides).
i12	I know the impact that biological products have on food-related sustainability (e.g., less soil contamination).
F3: Selection and planning	
i14	I control the calories and/or other nutritional characteristics of the food I eat daily.
i17	I read and interpret food labels to select the most appropriate foods.
i18	I make informed food choices.
i19	I dedicate time and invest in food selection (e.g., when I go shopping).
i22	I can easily substitute one food for another nutritionally equivalent one.
i23	I plan various aspects of my diet.
i24	I plan my meals in advance.
F4: Environmentally safe	
i9	I apply food hygiene and safety practices (e.g., storing food at appropriate temperatures, cleaning utensils).
i13	I buy local/national trade products to support local/national business.
i20	I eat food according to its seasonality.
i21	I am aware of the time of year of each food.
F5: Origin	
i15	I can identify the origin of a food (that is, where a food comes from).
i16	I can identify how a food is produced and processed (that is, how it is manufactured, how it is packaged).
