# Supplementary material for: Adaptation of the Food Literacy (FOODLIT) Tool for Turkish Adults: A Validity and Reliability Study
Source: Nutrients. 2024 Oct 9;16(19):3416. doi: 10.3390/nu16193416 (PMC11478845; doi:10.3390/nu16193416)
Supplement: Supplementary file 1 [file nutrients-16-03416-s001.zip › Supplemantary Material File S2.pdf]

GIDA OKURYAZARLIĞI ARACI TÜRKÇE VERSİYONU

Değerli katılımcı, lütfen her bir maddeyi dikkatlice okuyunuz ve size uygun gelen seçeneği işaretleyiniz.

1. Yemek yapmak için gereken her şeyi kolaylıkla hazırlarım.
0. Asla 1. Bazen 2. Sıklıkla 3. Her zaman

2. İyi bir yemek yapmak için farklı malzemeleri birleştiririm.
0. Asla 1. Bazen 2. Sıklıkla 3. Her zaman

3. Yemek tariflerini kendi damak tadıma göre uyarlarım.
0. Asla 1. Bazen 2. Sıklıkla 3. Her zaman

4. Mutfak araç ve gereçlerini (örn., fırını, mutfak robotunu) etkin bir şekilde kullanırım.
0. Asla 1. Bazen 2. Sıklıkla 3. Her zaman

5. Yeterli miktarda yemeği genellikle evde bulunan malzemelerle pişiririm.
0. Asla 1. Bazen 2. Sıklıkla 3. Her zaman

6. Farklı pişirme tekniklerini kullanırım. (örn., tencere yemeği, fırında pişirme)
0. Asla 1. Bazen 2. Sıklıkla 3. Her zaman

7. Yemek yapmaktan keyif alırım.
0. Asla 1. Bazen 2. Sıklıkla 3. Her zaman

8. Farklı gıdalara uygun farklı saklama yöntemleri (örn., dondurma, tuzlama) hakkında bilgim vardır.
0. Tamamen katılmıyorum 1. Katılmıyorum 2. Katılıyorum 3. Tamamen katılıyorum

9. Gıda hijyeni ve güvenliği ilkelerini uygularım (örn., gıdaları uygun sıcaklıkta saklamak, mutfak aletlerini temizlemek).
0. Asla 1. Bazen 2. Sıklıkla 3. Her zaman

10. Pestisitlerin ve/veya herbisitlerin (tarım ilaçları) gıdalar üzerindeki etkilerinin farkındayım.
0. Tamamen katılmıyorum 1. Katılmıyorum 2. Katılıyorum 3. Tamamen katılıyorum

11. Organik ürünlerin (örn., pestisit kullanılmadan yetiştirilen gıdalar) ne olduğunu bilirim.
0. Tamamen katılmıyorum 1. Katılmıyorum 2. Katılıyorum 3. Tamamen katılıyorum

12. Biyolojik ürünlerin gıda sürdürülebilirliği üzerindeki etkisini bilirim (örn., daha az toprak kirliliği).
0. Tamamen katılmıyorum 1. Katılmıyorum 2. Katılıyorum 3. Tamamen katılıyorum

13. Yerel/ulusal üretimi desteklemek için yerel/ulusal ürünleri satın alırım.
0. Asla 1. Bazen 2. Sıklıkla 3. Her zaman

14. Gün içerisinde yediklerimin enerji değerini ve/veya diğer besinsel özelliklerini kontrol ederim.
0. Asla 1. Bazen 2. Sıklıkla 3. Her zaman

15. Bir gıdanın menşeini (nereden geldiğini) tanımlayabilirim.
0. Asla 1. Bazen 2. Sıklıkla 3. Her zaman

16. Bir gıdanın nasıl üretildiğini ve işlendiğini (nasıl imal edildiğini ve paketlendiğini) tanımlayabilirim.
0. Asla 1. Bazen 2. Sıklıkla 3. Her zaman

17. En uygun gıdayı seçmek için gıda etiketlerini okur ve yorumlarım.
0. Asla 1. Bazen 2. Sıklıkla 3. Her zaman

18. Gıda seçimlerimi bilinçli olarak yaparım.
0. Asla 1. Bazen 2. Sıklıkla 3. Her zaman

19. Gıda seçimine zaman ve bütçe ayırırım (örn., alışverişe gittiğimde).
0. Asla 1. Bazen 2. Sıklıkla 3. Her zaman

20. Gıdaları mevsimine uygun tüketirim.
0. Asla 1. Bazen 2. Sıklıkla 3. Her zaman

21. Her bir gıdanın yıl içerisinde yetiştiği/tüketilebileceği zamanın (mevsiminin) farkındayım.
0. Tamamen katılmıyorum 1. Katılmıyorum 2. Katılıyorum 3. Tamamen katılıyorum

22. Bir gıda yerine besin değeri açısından eşdeğer olan bir başka gıdayı kolayca koyabilirim.
0. Tamamen katılmıyorum 1. Katılmıyorum 2. Katılıyorum 3. Tamamen katılıyorum

23. Günlük diyetimi (beslenmemi) çeşitli yönleriyle planlarım.
0. Asla 1. Bazen 2. Sıklıkla 3. Her zaman

24. Öğünlerimi önceden planlarım.
0. Asla 1. Bazen 2. Sıklıkla 3. Her zaman

Faktörler

F1: Mutfak Becerileri (1, 2, 3, 4, 5, 6, 7 ve 8. madde)

F2: Üretim ve Kalite (10, 11, 12. madde)

F3: Seçim ve Planlama (14, 17, 18, 19, 22, 23, 24. madde)

F4: Çevreye Duyarlılık (9, 13, 20, 21. madde)

F5: Menşei (15, 16. madde)
